# Finite Element Analysis of Protective Measures against Lateral Hinge Fractures in High-Tibial Osteotomy

**DOI:** 10.1155/2024/5510319

**Published:** 2024-08-22

**Authors:** Emre Özmen, Alican Baris, Esra Circi, Serdar Yuksel, Ozan Beytemür

**Affiliations:** ^1^ Istanbul Physical Treatment and Rehabilitation Training and Research Hospital, Istanbul, Türkiye; ^2^ SBU Bagcilar Training and Research Hospital, Istanbul, Türkiye

## Abstract

**Background:**

Opening wedge high-tibial osteotomy (OWHTO) is widely used for correcting mechanical axis deviations and offloading the medial compartment in unicompartmental osteoarthritis. However, lateral hinge fractures (LHFs) pose a significant complication. This study investigates protective measures to mitigate these fractures, guided by prior observations of mechanical stress impact on LHFs.

**Purpose:**

The study aims to assess the effectiveness of different protective measures, specifically the use of varying sizes of Kirchner wires and drill holes, in reducing the incidence of LHFs during OWHTO. *Study Design*. The study employs a quantitative, comparative analysis using a finite element method (FEM) based on computed tomography (CT) scans.

**Methods:**

Using CT-based FEM, the study compares the impact of different sizes of K-wires (1.6 mm, 2.0 mm, and 2.5 mm) and drill holes (3.2 mm and 4.5 mm) on the mechanical stresses around the hinge area in OWHTO. The models were created from a CT scan of a healthy 33-year-old male, focusing on the force required to open the osteotomy gap and the incidence of cracked shell elements.

**Results:**

The study found that thicker K-wires increased the force required to open the osteotomy gap, whereas larger apical holes decreased it. The 4.5 mm apical hole model demonstrated significantly fewer cracks compared to the 2.0 mm K-wire model, with no significant difference observed compared to the 2.5 mm K-wire model. Models using a 1.6 mm K-wire or a 3.2 mm drill hole did not significantly reduce cracks compared to the base model.

**Conclusions:**

The findings suggest that a 4.5 mm drill hole may be more effective in reducing the risk of LHFs compared to thinner diameter K-wires or smaller apical holes. Both a 2.5 mm K-wire and a 4.5 mm drill hole reduce the number of cracked elements, but the 4.5 mm drill hole also significantly decreases the average and maximum principal stresses as well as the average tensile strength ratio at the hinge area. These findings may be important for surgical planning, particularly in cases requiring increased osteotomy distraction.

## 1. Introduction

Opening wedge high-tibial osteotomy (OWHTO) of the proximal tibia is a highly effective technique for correcting deviations in the mechanical axis as well as offloading the medial compartment in cases of unicompartmental osteoarthritis (OA) [1, 2].

The goal is usually to have a mechanical axis that passes from the center of the knee or slightly lateral in cases where overcorrection is warranted [3]. Depending on the surgical goal, preoperative planning dictates the amount of the opening wedge.

One of the complications of OWHTO is the lateral hinge fractures [4, 5]. Fracture line in this situation can be an extension of the osteotomy, extending in a distal direction to the hinge or in an opposite manner reach to the articular surface. These fracture types have been classified as type 1, 2, and 3 LHFs by Takeuchi et al. [6].

A couple of risk factors may contribute to the occurrence of LHFs such as opening the wedge too fast, improper hinge position, or insufficient osteotomy [7–9].

Since the biomechanical reason behind hinge fractures are ultimately abnormal tensile and compressive stresses around the hinge, different protective measures have been suggested to protect against this complication. One of the proposed solutions is the “golden pin” or “golden Kirschner wire (K-wire),” which refers to a K-wire inserted distal to the osteotomy site aiming proximally in an oblique fashion to stabilize the hinge [10]. Another proposed solution is drilling the apex of the hinge in an anteroposterior fashion to decrease stresses at the hinge point [11, 12]. The finite element method (FEM) is a numerical method to study deformations and fractures in different loading conditions in biomechanics. Computed tomography (CT)-based FEM enables accurately modeling the properties of the studied bone through using hydroxyapatite phantoms or conversion formulas [13].

This study aims to evaluate the effectiveness of various sizes of protective K-wires and apical drill holes in minimizing the risk of lateral hinge fractures in OWHTO.

## 2. Materials and Methods

**Figure 1:**
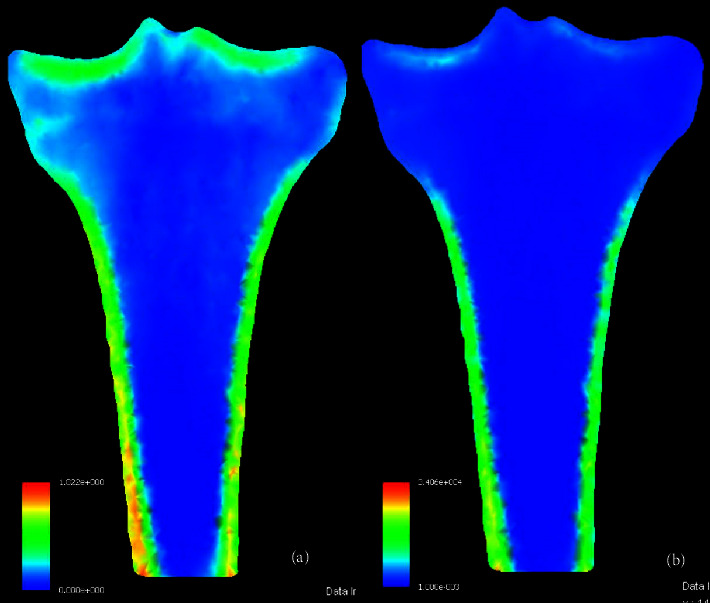
(a) Density (mg/mm^3^) and (b) Young's modulus (MPa) of the tibia model after conversion of CT values through Keyak's formula.

Institutional Review Board approval was obtained for this study. A CT scan of a 33-year-old healthy male was used for FEA. The CT scan slices were segmented semiautomatically. Six different 3D-models with a 2.0 mm mesh size [14] with triangular elements on the outer surface and tetrahedral elements for the inner volume was created using Mechanical Finder version 12.0 (Research Center for Computational Mechanics, Tokyo, Japan). To create a heterogeneous model, the material properties of the bone were calculated from CT density values using conversion formula by Keyak [13, 15] (Figures 1(a) and 1(b)). Poisson's ratio for the bone was set at 0.4.

A 1.26 mm thick rectangular chisel with a width of 26 mm and length of 100 mm was designed in SpaceClaim R17.1 (SpaceClaim Corporation, USA) to represent the bone loss with the saw cut. Similarly, 1.6 mm, 2.0 mm, and 2.5 mm K-wires and well as cylindrical models representing drill holes by 3.2 and 4.5 mm drill bits were modeled.

An osteotomy was performed by intersecting the chisel model with the bone model along the desired osteotomy plane and direction, aimed towards the tip of the fibular head, and terminating 1 cm distal to the joint line and medial to the lateral cortex [8]. The intersecting area was then deleted from the model to simulate the osteotomy.

**Figure 2:**
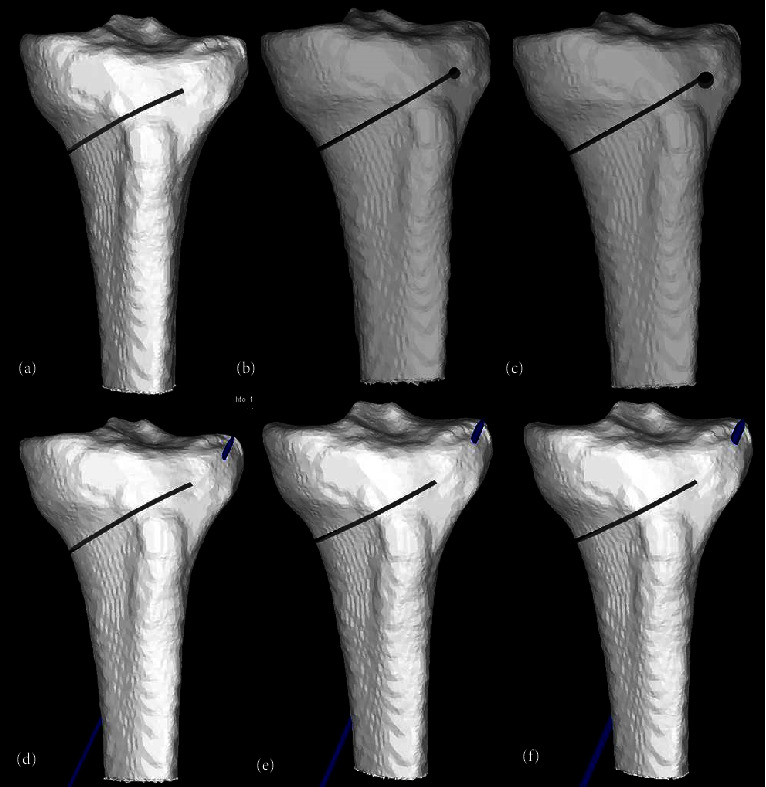
Models used in the study. (a) Base uniplanar opening wedge HTO model (b) 3.2 mm apical hole model (c) 4.5 mm apical hole model (d) 1.6 mm K-wire model (e) 2.0 mm K-wire model (f) 2.5 mm K-wire.

Once the base model (osteotomy without protective measures) was created, different sized drill holes were introduced similarly. Cylinders with diameters of 3.2 mm and 4.5 mm were placed at the apex of the osteotomy in a sagittal orientation, and the intersecting areas were deleted from the model. To simulate protective K-wires, cylinders with diameters of 1.6 mm, 2.0 mm, and 2.5 mm were placed at the same angle and direction (Figure 2). The wires were assigned material properties representing surgical stainless steel (Poisson's ratio = 0.3 and Young's modulus = 193 Gpa) [16].

**Figure 3:**
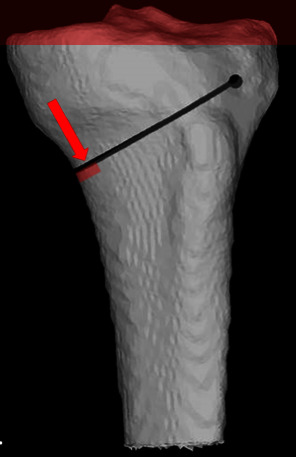
Loading (area around the tip of the red arrow) and boundary conditions (2 mm of proximal tibia from the joint line) are shown. The red arrow represents the direction of the tangential distractive force at the osteotomy site.

Boundary conditions were set such that tibia was fixed at the joint line and distractive force was applied to a cortical rim at the medial part of the osteotomy site (Figure 3)

Different forces applied to the osteotomy site using finite element analysis to distract the osteotomy site 10–15 mms. Each osteotomy gap analysis was done separately. The FEA was carried out under the assumption of linear static loading conditions. The Drucker–Prager criterion for the yield of each element was used. A region of interest (ROI) was selected around the hinge point, which was the same for all models. Maximum and average principal stresses as well as number of cracked shell elements were extracted from the ROI. The tensile strength ratio (maximum principal stress/critical stress) was calculated for each node in the ROI. SPSS Statistics Version 20.0 (IBM, Chicago, IL) was used for statistical analysis. A *P* value less than 0.05 was accepted as significant. The Shapiro–Wilk test was used to test normality of the data. Levene's test was used to test homogeneity of variance. The ANOVA test was used to compare data among multiple groups.

## 3. Results

The base model, 1.6 mm and 2.0 mm 2.5 K-wire models and 3.2 mm and 4.5 mm drill bit models were compared according to force required to open the osteotomy gap, cracked elements, average and maximum principle stresses, and average tensile strength ratios (maximum principal/critical stress percentages) around the ROI.

**Figure 4:**
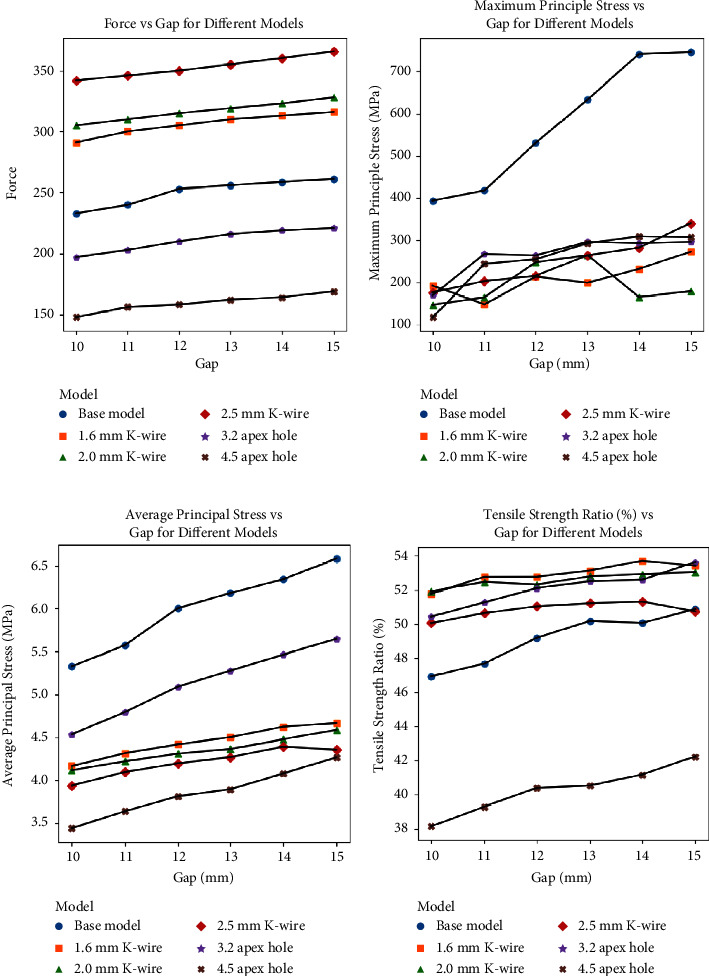
(a) Force required to open the osteotomy site vs. gap. (b) Maximum principal stress vs. gap. (c) Average principal stress vs. gap. (d) Tensile strength ratio vs. gap.

Force required to open the osteotomy gap for a given amount increased with thicker K-wire compared to the base model and decreased with a larger apical hole (Figure 4(a)). The ANOVA test revealed a highly significant difference in force measurements across the different models (*p* < 0.0001). Post hoc analysis with Tukey's HSD test was done to assess significance across models pairwise and revealed specific pairwise differences among the models. The force required to open the osteotomy between models utilizing 1.6 mm K-wire and 2.0 mm K-wire did not exhibit statistically significant variations (*p* > 0.05). The other pairs had significant pairwise differences (*p* < 0.01)

Percent of cracked shell elements (Table 1) were significantly different across models according to the ANOVA test (*p* < 0.0001). Subsequent post hoc analysis using Tukey's HSD revealed specific pairwise differences between models. The “Base model” and the “2.0 mm K-wire” model, the “base model” and the “2.5 mm K-wire” model, as well as the “base model” and the “4.5 apex hole” model showed significant differences in their mean crack percentages, with the “base model” having higher crack percentages compared to the “2.0 mm K-wire,” “2.5 mm K-wire,” and “4.5 apex hole” models.

Interestingly, no significant difference was found between the “base model” and the “1.6 mm K-wire” model and the “base model” and the “3.2 apex hole” model, suggesting similar crack percentages between these pairs. Among 2.0 mm K-wire, 2.5 mm K-wire and 4.5 apical hole models, the 4.5 apical hole model had significantly fewer cracks compared to the 2.0 mm K-wire model but not significantly fewer compared to the 2.5 mm K-wire model.

The models were also compared for average and maximum principal stress (Figures 4(b) and 4(c)) and tensile strength ratio (maximum principal stress/critical stress) (Figure 4(d)).

In terms of maximum principal stress, the base model had the highest maximum principal stresses across the ROI (*p* < 0.001). Post hoc tests showed that the other models were similar to each other in terms of maximum principal stress. In terms of average principal stress in the ROI, the base model again had the highest average principal stresses (*p* < 0.001), 3.2 mm drill hole model had lower average principle stresses compared to the base model (*p* < 0.01), while the rest of the models had similarly lower levels of average principle stress compared to both (*p* < 0.01). The average tensile strength ratio (%) was also different across the models significantly. Surprisingly, the base model had a lower tensile strength ratio on average than 1.6 mm, 2.0, and 2.5 mm K-wire and 3.2 mm drill hole (*p* < 0.01), which all had higher average ratios. The 4.5 mm drill hole model had significantly lower measurements than all other models (*p* < 0.0001).

## 4. Discussion

Lateral hinge fractures are a significant complication of HTOs [17, 18]. When they are spotted intraoperatively, an intervention to stabilize the osteotomy and/or the joint line is at least possible. If they happen postoperatively, they might significantly hinder the healing process and rehabilitation.

This study aimed to evaluate the effectiveness of different protective measures against LHFs in OWHTO. The results indicated that both a 2.5 mm K-wire and a 4.5 mm drill hole significantly reduce the number of cracked elements compared to the base model, with the 4.5 mm drill hole offering additional benefits by reducing both the average and maximum principal stresses, as well as the average tensile strength ratio at the hinge area.

**Figure 5:**
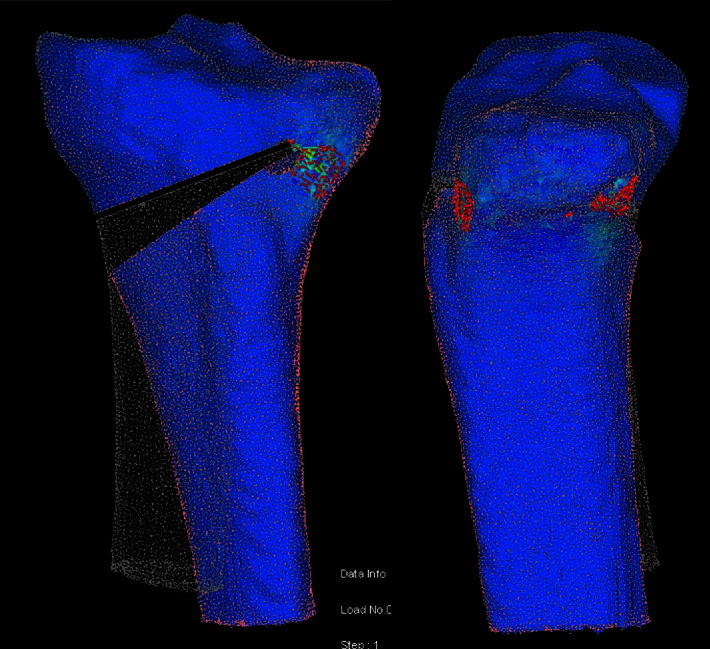
Anteroposterior and lateral views of a model. Note that the surface cracks do not cover join on the lateral side, representing to an osteotomy without hinge fracture.

The force required to open the osteotomy gap varied with the size of the K-wire and the drill bit. Thicker K-wires increased the necessary force, whereas larger apical holes reduced it. Statistically significant differences were observed across models in terms of force measurements, cracked shell elements, and crack percentages around the hinge (Figure 5). The 4.5 mm apical hole model exhibited fewer cracks compared to the 2.0 mm K-wire model but was statistically similar to the 2.5 mm K-wire model.

The findings of this study agree with previous research highlighting the importance of stabilizing the lateral hinge to prevent fractures. Dessyn et al. demonstrated that adding a protective K-wire during OWHTO significantly increases the lateral hinge's resistance to fracture [19]. Their cadaveric study showed that the maximum load to hinge breakage and the maximum permissible displacement were significantly higher in knees with K-wires compared to those without. This supports our findings that thicker K-wires, such as the 2.5 mm variant, provide substantial mechanical stability to the lateral hinge. Similarly, Gulagaci et al. reported that positioning a K-wire to intersect the osteotomy plane at the theoretical lateral hinge location reduced the occurrence of perioperative hinge fractures [7]. Their clinical study found that the lateral hinge fracture rate was significantly lower in patients with an additional K-wire compared to those without (16.7% vs. 43.3%). This clinical evidence corroborates our finite element analysis, suggesting that protective K-wires effectively mitigate hinge fractures during OWHTO.

Kang et al. highlighted the importance of stress distribution around the hinge point, suggesting that modifications to the osteotomy technique could improve outcomes by reducing peak stresses [20]. In similar vein, our study found that a 4.5 mm apical drill hole was particularly effective in reducing the risk of LHF. The tensile strength ratio, a measure that compares the maximum principal stress to the critical stress at a specific node, offers a more detailed understanding of fracture risk under stress than simply looking at the maximum and average principal stresses alone. While all models were an improvement compared to the base model in terms of maximum and average principle stresses, the average tensile strength ratio was higher in these models compared to the base model, indicating that the nodes were closer to their tensile failure point. On the other hand, the 4.5 mm model had significantly lower tensile strength ratio on average, which may suggest that it may be the model that puts the least stress on the hinge area. The significant reduction in principal stresses observed with the 4.5 mm drill hole in our study supports the idea that strategic modifications can enhance hinge stability.

This study, while providing valuable insights into the prevention of lateral hinge fractures in OWHTO, has certain limitations. First, the use of a finite element model is a numerical simulation and may not fully replicate the complex biomechanics of a real human knee. The model was based on the CT scan of a single, healthy 33-year-old male, which limits its generalizability across a diverse patient population with varying ages, bone densities, and health conditions. In addition, the study focused solely on mechanical aspects and did not account for biological factors such as bone healing and individual patient variability in response to surgery since we know that hinge fractures can happen postoperatively as well [21]. The protective measures evaluated, though promising, were limited to specific sizes of K-wires and drill holes, and their effectiveness might vary with different configurations or in clinical practice. These limitations highlight the need for further research, including clinical trials, to validate these findings and expand the understanding of LHF prevention in high-tibial osteotomy.

## 5. Conclusion

In conclusion, this study provides a detailed quantitative analysis of protective measures aimed at minimizing intraoperative LHFs in OWHTO. The findings indicate that both a 2.5 mm K-wire and a 4.5 mm drill hole significantly reduce the number of cracked elements compared to the base model. However, the 4.5 mm drill hole offers additional benefits by significantly decreasing both the average and maximum principal stresses, as well as the average tensile strength ratio at the hinge area. These results suggest that the use of a 4.5 mm drill hole may be more effective in reducing the risk of LHFs during OWHTO.

## Figures and Tables

**Table 1:** Number and percent of cracked shells for each model at each osteotomy gap.

Osteotomy gap (mm)	10		11		12		13		14		15	
Model	Cracked shells											
	*n*	%	*n*	%	*n*	%	*n*	%	*n*	%	*n*	%
Base	739	4.3	767	4.5	815	4.7	851	4.9	867	5.0	843	4.9
1.6 mm K-wire	643	3.8	716	4.3	729	4.4	760	4.5	750	4.5	755	4.5
2.0 mm K-wire	609	3.6	609	3.6	645	3.8	665	3.9	704	4.1	714	4.2
2.5 mm K-wire	565	3.4	589	3.6	599	3.6	611	3.7	625	3.8	676	4.1
3.2 mm apical hole	712	4.2	727	4.3	721	4.3	754	4.5	772	4.6	805	4.8
4.5 mm apical hole	501	3.0	534	3.2	558	3.4	575	3.5	610	3.7	646	3.9

## Data Availability

The data that support the findings of this study are available on request from the corresponding author. The data are not publicly available due to privacy or ethical restrictions.
